# Evodiamine suppresses endometriosis development induced by early EBV exposure through inhibition of ERβ

**DOI:** 10.3389/fphar.2024.1426660

**Published:** 2024-08-01

**Authors:** Junling Wang, Yuanqi Liang, Xiaoru Liang, Huijuan Peng, Yongxia Wang, Mingtao Xu, Xuefang Liang, Helen Yao, Xiaohan Liu, Liqin Zeng, Paul Yao, Dongfang Xiang

**Affiliations:** ^1^ Department of Gynecology, The Second Affiliated Hospital of Guangzhou University of Chinese Medicine, Guangzhou, China; ^2^ The Second Clinical College of Guangzhou University of Chinese Medicine, Guangzhou, China; ^3^ University of California at Riverside, Riverside, CA, United States; ^4^ Department of Gynecology, Sun Yat-Sen University Affiliated No. 8 Hospital, Shenzhen, China

**Keywords:** endometriosis, Epstein-Barr virus, estrogen receptor β, evodiamine, latent membrane protein 1

## Abstract

**Introduction:** Endometriosis (EMS) is characterized as a prevalent gynecological inflammatory disorder marked by the existence of endometrial tissues situated beyond the uterus. This condition leads to persistent pelvic pain and may contribute to infertility. In this investigation, we explored the potential mechanism underlying the development of endometriosis (EMS) triggered by transient exposure to either latent membrane protein 1 (LMP1) or Epstein-Barr virus (EBV) in a mouse model. Additionally, we examined the potential inhibitory effect of evodiamine (EDM) on EMS.

**Methods:** Immortalized human endometrial stromal cells (HESC) or epithelial cells (HEEC) were transiently exposed to either EBV or LMP1. The presence of evodiamine (EDM) was assessed for its impact on estrogen receptor β (ERβ) expression, as well as on cell metabolism parameters such as redox balance, mitochondrial function, inflammation, and proliferation. Additionally, a mixture of LMP1-treated HESC and HEEC was administered intraperitoneally to generate an EMS mouse model. Different dosages of EDM were employed for treatment to evaluate its potential suppressive effect on EMS development.

**Results:** Transient exposure to either EBV or LMP1 triggers persistent ERβ expression through epigenetic modifications, subsequently modulating related cell metabolism for EMS development. Furthermore, 4.0 µM of EDM can efficiently reverse this effect in *in vitro* cell culture studies. Additionally, 20 mg/kg body weight of EDM treatment can partly suppress EMS development in the *in vivo* EMS mouse model.

**Conclusion:** Transient EBV/LMP1 exposure triggers permanent ERβ expression, favoring later EMS development, EDM inhibits EMS development through ERβ suppression. This presents a novel mechanism for the development of endometriosis (EMS) in adulthood stemming from early Epstein-Barr virus (EBV) exposure during childhood. Moreover, evodiamine (EDM) stands out as a prospective candidate for treating EMS.

## Introduction

Endometriosis (EMS) is defined as a prevalent inflammatory condition affecting the female reproductive system identified by the existence of lesions resembling endometrial tissues situated beyond the uterus. This condition results in chronic pelvic pain, infertility, and adversely impacts the overall quality of life for affected women (Zondervan et al., 2018). Currently, the treatment for EMS relies on surgical removal and hormone suppression (Bulun et al., 2019), which includes Gonadotropin-releasing hormone (GnRH) antagonists and progestins. However, hormone suppression often entails numerous side effects (Zeng et al., 2024). Endometriosis (EMS) exhibits estrogen dependence, featuring a substantial upregulation of estrogen receptor β (ERβ). ERβ and the genes it regulates (Xiang et al., 2020), play a pivotal role in EMS pathogenesis, influencing redox balance (Liu et al., 2014; Kong et al., 2016; Zhan et al., 2016), apoptosis, inflammation (Han et al., 2015; Simmen and Kelley, 2016; Han et al., 2019). However, the specific mechanism leading to the upregulation of ERβ in EMS remains largely unknown.

Many factors may contribute to EMS development, including retrograde menstruation, modulation of metabolic pathways and the endocrine system, epithelial-mesenchymal transition, as well as altered immunity, inflammation, and redox balance (Wang et al., 2020; Xiao et al., 2020; Bulun, 2022). Some studies suggest a potential link between EMS and virus infection, with Moslehi et al. (2023) recently reporting a possible correlation between human papillomavirus infection and endometriosis. However, the overall association of viruses with EMS is still regarded as relatively low (Vestergaard et al., 2010). Epstein-Barr virus (EBV), a γ-herpes virus, is linked to various human tumors, in part, due to its role in the transformation of B-cell lymphocytes and the presence of latent membrane protein 1 (LMP1) (Chou et al., 2011; Wang et al., 2017). Approximately ∼95% of the population is infected by EBV in their lives, while most of them experience latent infection with no obvious symptoms (Hu et al., 2016; Wang et al., 2019). Recently, our research has revealed that EBV contributes to tumorigenesis by upregulating peroxisome proliferator-activated receptor gamma coactivator 1-β (PGC1β) through the action of LMP1 (Feng et al., 2021). Furthermore, transient EBV infection initiates persistent expression of PGC1β during later stages of tumor development (Chen et al., 2023). We assume that EBV or LMP1 early exposure may trigger persistent ERβ upregulation and contribute to later endometriosis development.

Evodiamine (EDM), a natural alkaloid ingredient with a quinazolinocarboline skeleton structure, can be extracted from the fruit of Evodiae rutaecarpa (Xu et al., 2022; Zhang et al., 2023). It possesses various biological benefits, including anti-angiogenesis (Shyu et al., 2006; Yu et al., 2023), anti-tumor growth (Wang et al., 2013; Shi et al., 2016), promotion of apoptosis (Li et al., 2022), modulation of mitochondrial function (Liu et al., 2013), oxidative stress (Yang et al., 2008), and anti-inflammatory effects (Yang et al., 2021). In this study, we hypothesize that EDM may have potential effects on EMS development due to its modulation activities on inflammation, proliferation, apoptosis, and redox balance (Wang et al., 2013; Xu et al., 2022).

In our earlier research, we identified an association between endometriosis and ERβ upregulation, leading to the initiation of endometriosis development (Xiang et al., 2020). Nonetheless, the underlying mechanism responsible for the upregulation of ERβ in endometriosis remains elusive. In this investigation, our aim is to examine the possible mechanism behind the sustained expression of ERβ triggered by exposure to EBV or LMP1. Additionally, we aim to assess the inhibitory effect of evodiamine (EDM) on ERβ expression and its subsequent impact on suppressing endometriosis. Human endometrial stromal cells (HESC) or epithelial cells (HEEC) were immortalized and exposed to either EBV or LMP1 adenovirus transiently. Following the removal of EBV or LMP1, we monitored the expression of ERβ and its subsequent molecular properties related to proliferation, mitochondrial function, redox balance and pro-inflammatory cytokine release in an *in vitro* cell culture study. Also, we assessed the potential inhibitory impact of EDM on the expression of ERβ. Additionally, a mixture of EBV/LMP1 exposure-mediated HESC and HEEC cells was administered intraperitoneally to generate an endometriosis mouse model (Banu et al., 2008; Arosh et al., 2015; Arosh et al., 2022), and the inhibitory effect of EDM on endometriosis development was evaluated.

## Materials and methods

For additional details on the *Materials and methods*, please refer to Supplementary Data S1.

### Reagents and materials

Human endometrial epithelial cells (HEEC, #ABC-H0045X) and endometrial stromal cells (HESC, #ABI-TM257D) were immortalized by SV40 large T antigen, purchased from AcceGen (Beijing, China), and cultured in medium using the ABC-TM045X Human Endometrial Epithelial Cells Medium Kit and ABI-TM257D Human Endometrial Stromal Cell Line (HESC) Medium Kit, respectively (from AcceGen). Evodiamine (EDM), >99% purity, with a molecular formula of C19H17N3O and a molecular weight of 303.36, was obtained from Wuhan Ability Chemical Technology, China, and dissolved in dimethyl sulfoxide (DMSO, Sigma China) with 0.1% as the final working solution (Yu et al., 2023). ERβ antagonist PHTPP (#2662) was obtained from Tocris Bioscience, and dissolved in DMSO (0.1% as the final).

### Methods

The quantification of mRNA levels was conducted through real-time qPCR, utilizing primers specified in Supplementary Table S1, while protein levels were assessed through Western blotting and immunostaining. Cell viability was determined using MTT assay (Yao et al., 2009). Histone methylation was analyzed by chromatin immunoprecipitation (ChIP) using primers from Supplementary Table S1 (Zhang et al., 2017; Zou et al., 2017). Reactive oxygen species (ROS) was assessed by measuring CM-H2DCFDA-mediated fluorescence emission using a microplate reader at 485/530 nm (Zhang et al., 2017; Zou et al., 2017). The GSH/GSSG ratio was assessed through GSH/GSSG-Glo™ Assay Kit. The measurement of 3-nitrotyrosine (3-NT) formation was conducted using a 3-Nitrotyrosine ELISA Kit and γH2AX formation was determined by Western blotting (Zhang et al., 2017). Mitochondrial function was assessed by quantifying mitochondrial DNA copies using qPCR, ATP levels (Yao et al., 2005; Zou et al., 2017; Zhang et al., 2018), and mitochondrial membrane potential (MMP, Δψm) by measuring TMRE-based fluorescence at 548/573 nm (Kong et al., 2016; Zhang et al., 2017; Zhang et al., 2018). Apoptosis was determined through TUNEL assay through *In Situ* Cell Death Detection Kit™ (Yao et al., 2005). EBV replication was determined by qPCR, quantifying EBV BMRF1 and normalizing by β-actin with primers from Supplementary Table S1 (Rose et al., 2002; Verma et al., 2016). Cytokines (IL1β, IL6, TNFα, and PGE2) were measured using the Human/Mouse Quantikine ELISA Kit from R&D Systems (Kobayashi et al., 2016). Cell proliferation was determined through the incorporation of [^3^H]-thymidine for DNA synthesis and the formation of colonies in soft agar (Zhang et al., 2017).

In order to evaluate the potential effects of EDM treatment and EBV/LMP1 exposure, the cells were separated into the following 5 groups for *in vitro* study. Group 1: cells were treated by empty adenovirus (EMP) together with control (CTL) solvent with 0.1% DMSO for 48 h (EMP/CTL); Group 2: cells were treated by EBV virus together with control (CTL) solvent with 0.1% DMSO for 48 h (EBV/CTL); Group 3: cells were treated by LMP1 adenovirus together with control (CTL) solvent with 0.1% DMSO for 48 h (LMP1/CTL); Group 4: cells were treated by EBV virus together with 4.0 µM of EDM dissolved in DMSO with 0.1% as the final working solution (EBV/EDM); Group 5: cells were treated by LMP1 adenovirus together with 4.0 µM of EDM dissolved in DMSO with 0.1% as the final working solution (LMP1/EDM).

### DNA methylation analysis

The evaluation of DNA methylation on the ERβ promoter was accomplished through methylation-specific PCR (MSP), coupled with bisulfite modification through the EpiJET Bisulfite Conversion Kit. Methylated and unmethylated primers were utilized for amplification, as follows: Methylated primer: forward 5′- gga tta tag gcg tga gtt att acg t -3′, reverse 5′- att taa aca caa aaa ttt aat cac gaa -3′; Unmethylated primer: forward 5′- gga tta tag gtg tga gtt att atg t -3′, reverse 5′- att taa aca caa aaa ttt aat cac aaa -3′. The product sizes were 210 bp (methylated) with Tm: 68.2°C and 210 bp (unmethylated) with Tm: 67.6°C. The ultimate methylation measurement was standardized based on the unmethylated input PCR (Eads et al., 2000; Ogino et al., 2006).

### *In vivo* mouse experiments

The NOD scid gamma (NSG) mouse was procured from Jackson Lab. The mouse protocol was approved by the Institutional Animal Care and Use Committee from Guangzhou University of Chinese Medicine following the NIH guidelines. At 4 weeks of age, sterile 60-day release pellets containing 0.72 mg of 17β-estradiol were implanted subcutaneously in all NSG female mice through a approximately 3-mm cut made on the dorsal side of the neck. Following 2 days of E2 pellet administration, the mixed human endometrial cells were used for transplantation.

#### Cell transplantation and EDM treatment

Immortalized HESC) and HEEC cells were subjected to infection with either empty control or LMP1 adenovirus for a duration of 48 h. Then, the adenovirus was removed and cultured continuously to pick up the single colony until passage 6. On the day of transplantation, LMP1 adenovirus or empty control (EMP)-treated immortalized HESC and HEEC cells were trypsinized, and 2 × 10^6^ cells for both HESC and HEEC cells were mixed, pelleted, and washed. The cells were then suspended and mixed with Matrigel at a 1:1 ratio to achieve a final 150 µL volume, maintaining the mixture on ice until transplantation. The blended suspensions were injected intraperitoneally using a 1 mL insulin syringe with a 20-gauge needle. The injection was administered along the midventral line, just caudal to the umbilicus, with care taken to avoid injury to the peritoneal layer or organs. Immediately post-transplantation, mice were kept in a sternal posture to facilitate cell attachment to the peritoneum (Banu et al., 2009). After 24 h of cell transplantation, the mice were randomly allocated into four groups and subjected to treatment with either EDM or a corresponding vehicle. Group 1: cells were pretreated by empty adenovirus (EMP) for cell transplantation, then mice received vehicle control (EMP/CTL). Group 2: cells were pretreated by LMP1 adenovirus for cell transplantation, then mice received vehicle control (LMP1/CTL). Group 3: cells were pretreated by LMP1 adenovirus for cell transplantation, then mice received 10 mg/kg body weight of EDM (EDM10) given as gavage every 2 days for 4 weeks (LMP1/EDM10). Group 4: cells were pretreated by LMP1 adenovirus for cell transplantation, then mice received 20 mg/kg body weight of EDM given as gavage every 2 days for 4 weeks (LMP1/EDM20) (Yu et al., 2023).

#### Assessment and characterization of endometriosis lesions

Following 4 weeks of cell transplantation, NSG mice recipients were humanely euthanized through CO_2_ asphyxiation, and whole blood was obtained via heart puncture. Peripheral blood mononuclear cells (PBMC) and serum were then isolated. The abdominal cavity was opened, and endometriosis lesions were visually examined for gross presence. The number of lesions was quantified using a dissection microscope, and the dimensions of each lesion were measured using a caliper. Lesions were categorized as single (comprising one visible nodule) or multiple (consisting of two or more visible nodules) (Banu et al., 2009; Arosh et al., 2015). A portion of the lesions was utilized for assessing the GSH/GSSG ratio, proinflammatory cytokine secretion, and gene expression (Gou et al., 2019). Simultaneously, the peritoneum was promptly fixed in 4% paraformaldehyde, and the entire peritoneum was sectioned for subsequent immunohistochemistry (IHC) staining (Banu et al., 2009).

### Statistical analysis

The data was expressed as mean ± SD (standard deviation), and each experiment was carried out in at least quadruplicate. The data was analyzed for normal distribution using the Shapiro-Wilk test. Statistical significance among different groups was determined using one-way analysis of variance (ANOVA) followed by the Tukey-Kramer test, with a significance level set at *p* < 0.05. All analyses were conducted using SPSS 22 software (Ramezani et al., 2022).

## Results

### Transient exposure to EBV/LMP1 results in persistent ERβ upregulation

**FIGURE 1:**
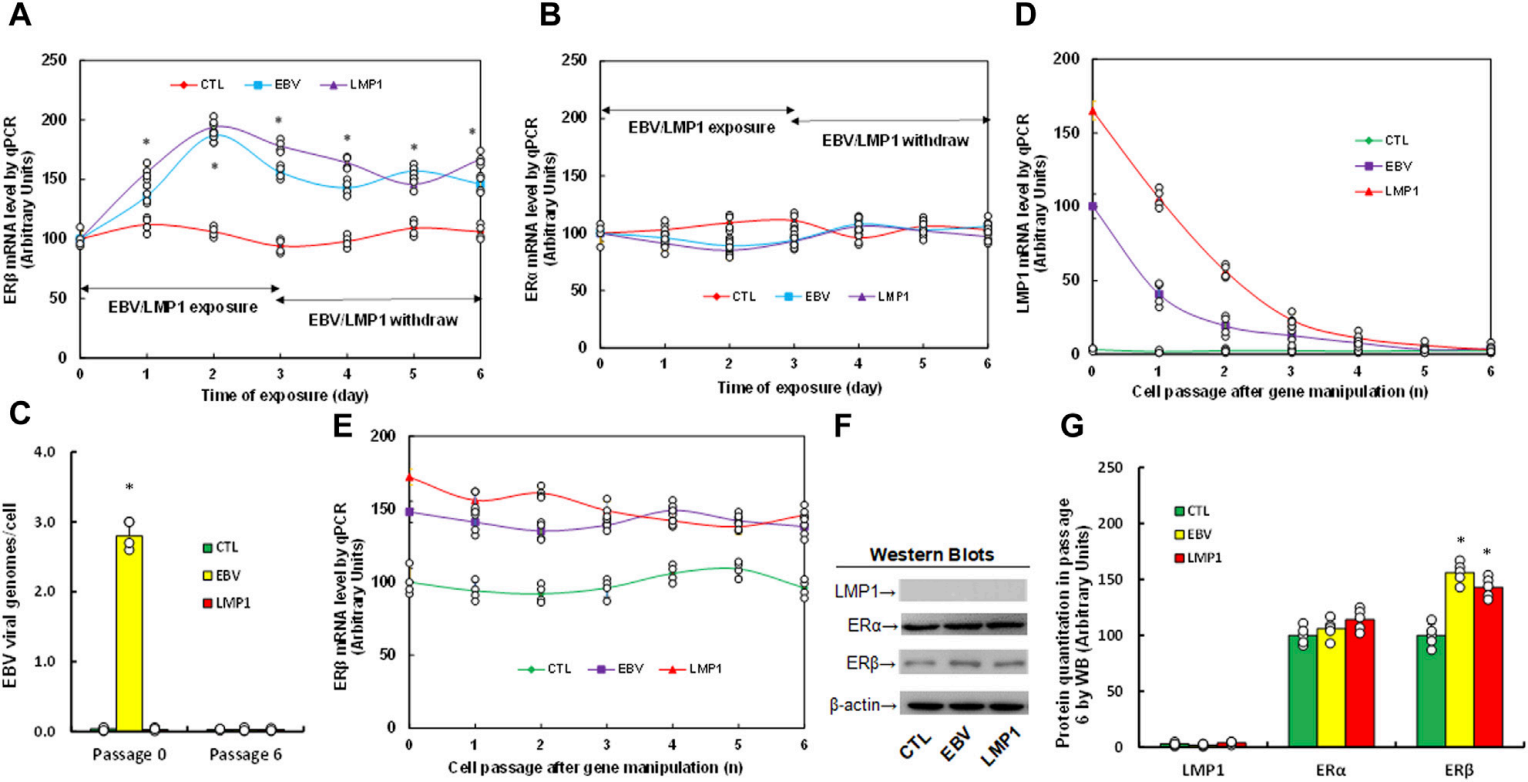
Transient exposure of EBV/LMP1 results in persistent ERβ upregulation. **(A,B)** Immortalized human HESC cells were treated by either control (CTL), EBV virus or LMP1 adenovirus for 3 days, then switched to control (CTL) for another 3 days, and the mRNA levels were determined for ERβ **(A)** and ERα **(B)**. n = 4. *p < 0.05, vs. day 0 treatment. **(C–G)** Immortalized human HESC cells were treated by either control (CTL), EBV virus or LMP1 adenovirus for 3 days (passage 0), then switched to CTL for continuous culturing until passage 6 for biological assays. **(C)** EBV viral genomes, n = 4, *p < 0.05, vs. CTL in Passage 0. **(D,E)** mRNA levels for LMP1 **(D)** and ERβ **(E)**, n = 4. **(F)** Representative western blots for **(G)**. **(G)** Protein quantitation at passage 6. n = 5, *p < 0.05, vs. CTL group. Data were expressed as mean ± SD, one-way ANOVA was used for statistical analysis. Abbreviations EBV, Epstein-Barr virus; ERα, estrogen receptor α; ERβ, estrogen receptor β; LMP1, latent membrane protein 1; WB, Western blotting.

We assessed the potential impact of infection with either EBV or LMP1 adenovirus on estrogen receptor expression. Immortalized HESC cells were exposed to treatment with either control (CTL), EBV virus, or LMP1 adenovirus for a period of 3 days. Following that, the cells were transitioned to CTL for an extra 3 days, and mRNA levels were then determined. We found that ERβ mRNA increased continuously following the infection of either EBV or LMP1, and the expression remained high after the removal of EBV/LMP1 (see Figure 1A). On the other hand, ERα mRNA showed no changes following virus infection (see Figure 1B). After the transient infection of EBV/LMP1 for 3 days (passage 0), the cells were cultured continuously until passage 6 following the removal of EBV/LMP1 for analysis. We found that EBV DNA copies were around 2.8 copies/cell after immediate EBV infection, while they completely disappeared at passage 6 (see Figure 1C). Additionally, LMP1 mRNA continuously decreased following the increase in cell passage and became completely undetectable at passage 6 (see Figure 1D). Conversely, ERβ mRNA levels demonstrated an increase following EBV/LMP1 infection compared to the CTL group, and this elevation persisted until passage 6 (see Figure 1E). Protein levels were also assessed at passage 6, revealing undetectable levels of LMP1. ERα exhibited no significant differences among the treatments, while ERβ protein levels remained elevated following transient exposure to EBV/LMP1 at passage 6 when compared to the CTL group (see Figures 1F, G; Supplementary Figure S1A). In conclusion, we found that transient exposure to EBV/LMP1 results in persistent ERβ upregulation even in the absence of EBV/LMP1.

### Evodiamine (EDM) inhibits the expression of ERβ by inducing epigenetic modifications

**FIGURE 2:**
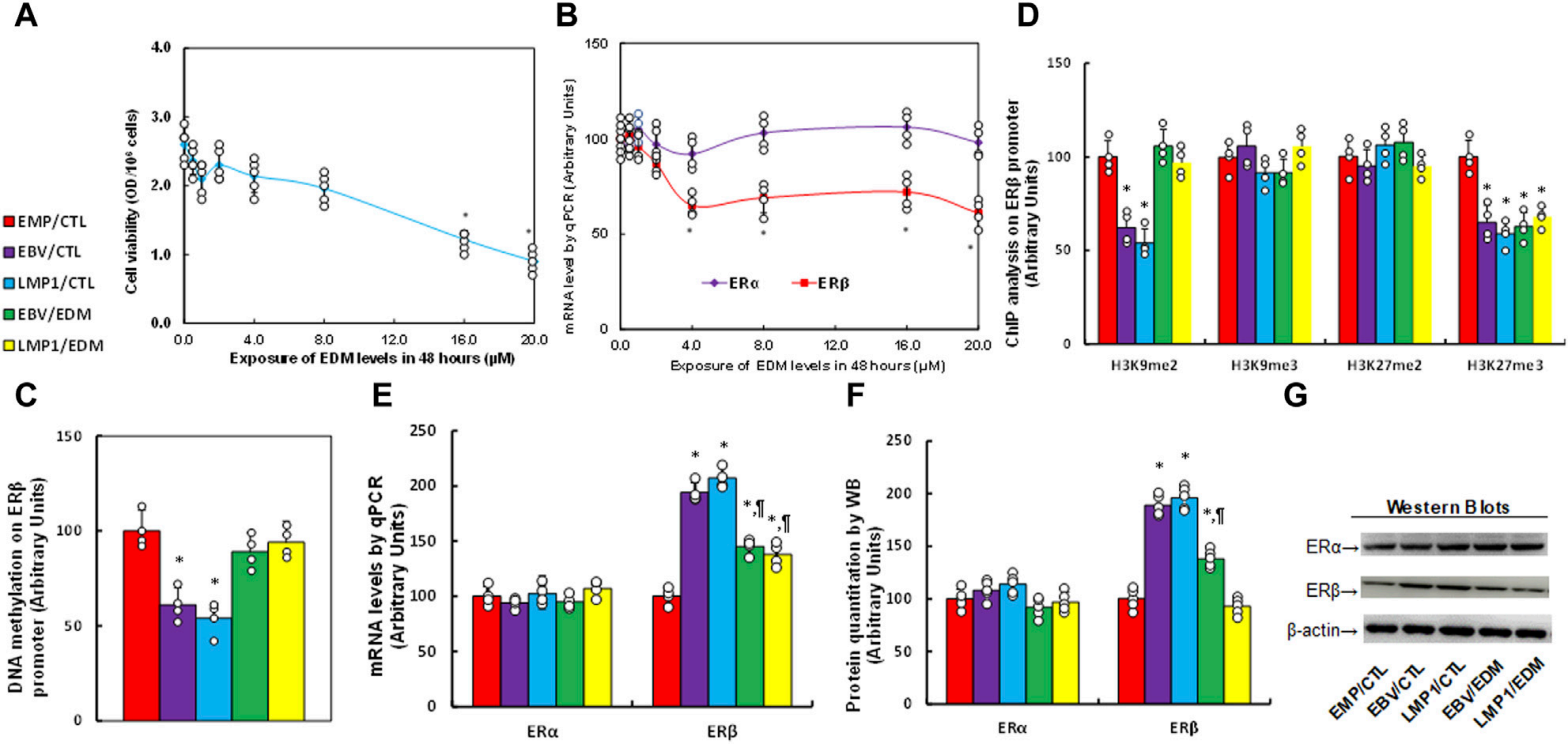
EDM suppresses ERβ expression through epigenetic modification on the ERβ promoter. **(A,B)** Immortalized human HESC cells were exposed to different concentrations of evodiamine (EDM), which including 0.0, 0.5, 1.0, 2.0, 4.0, 8.0, 12.0, 16.0 and 20.0 µM, after incubation of 48 h, cells were harvested for the analysis of cell viability **(A)** and mRNA levels **(B)**. n = 5, *p < 0.05, vs. 0.0 µM EDM. **(C–G)** Immortalized human HESC cells at passage 6 were pre-treated with empty (EMP), EBV or LMP1 adenovirus, then exposed to either control (CTL) or 4.0 µM of EDM for 48 h, then the cells were harvested for subsequent biological assays. **(C)** DNA methylation, n = 4. **(D)** ChIP analysis on the ERβ promoter, n = 4. **(E)** mRNA levels, n = 4. **(F)** Protein quantitation, n = 5. **(G)** Representative western blots for **(F)**. *p < 0.05, vs. EMP/CTL group; ¶*p* < 0.05, vs. EBV/CTL treatment. Data were expressed as mean ± SD, one-way ANOVA was used for statistical analysis. Abbreviations ChIP, chromatin immunoprecipitation; EBV, Epstein-Barr virus; EDM, evodiamine; ERα, estrogen receptor α; ERβ, estrogen receptor β; LMP1, latent membrane protein 1.

Immortalized human HESC cells were exposed to different concentrations of evodiamine (EDM) for 48 h for biological analysis, and we found that cell viability did not show a significant difference until the EDM concentration reached 16.0 µM (see Figure 2A). Additionally, the ERβ mRNA level started to decrease significantly when EDM concentration reached 4.0 µM, while ERα mRNA showed no difference among all the treatments (see Figure 2B). The cells were treated with 4.0 µM EDM in all the following *in vitro* cell culture experiments. The immortalized human HESC cells were pre-treated with empty (EMP), EBV, or LMP1 adenovirus, then further exposed to either control (CTL) or 4.0 µM of EDM for 48 h at passage 6 for subsequent biological assays. We observed a significant reduction in DNA methylation on the ERβ promoter following pre-treatment with either EBV or LMP1 in comparison to the EMP/CTL group, and the effect was completely reversed by EDM treatment (see Figure 2C). Examining histone methylation on the ERβ promoter showed that EBV/LMP1 pre-treatment led to a notable decrease in histone modifications of H3K9me2 and H3K27me3 in comparison to the EMP/CTL group, the effect was entirely reversed by EDM treatment on H3K9me2 modification, but had no impact on H3K27me3 modification. Moreover, no significant differences were observed in H3K9me3 and H3K27me2 among these treatments (see Figure 2D). Furthermore, mRNA levels in these cells were determined, indicating that EBV/LMP1 pre-treatment significantly increased ERβ expression compared to the EMP/CTL group. EDM treatment partially reversed this effect, while ERα expression showed no significant difference among the groups (see Figure 2E). Protein level evaluation mirrored the mRNA expression pattern (see Figures 2F, G; Supplementary Figure S1B). Furthermore, we evaluated the potential effect of EBV/LMP exposure on the ERβ activation, found that ERβ specific blockade PHTPP had no effect on EBV/LMP1 exposure-mediated ERβ upregulation (see Supplementary Figure S2A), while it completely diminished EBV/LMP1 exposure-mediated cell proliferation (see Supplementary Figure S2B). Additionally, we explored the potential impact of EBV/LMP1 pretreatment and EDM in HEEC cells. The results demonstrated that transient exposure to EBV/LMP1 significantly upregulated ERβ expression, and EDM treatment completely reversed this effect. ERα showed no significant difference among these groups (see Supplementary Figure S3A). Also, transient EBV/LMP1 exposure significantly reduced DNA methylation (see Supplementary Figure S3B), H3K9me2, and H3K27me3 (see Supplementary Figure S3C) on the ERβ promoter, and again, EDM treatment completely reversed this effect.

### EDM modulates EBV exposure-mediated oxidative stress in HESC cells

**FIGURE 3:**
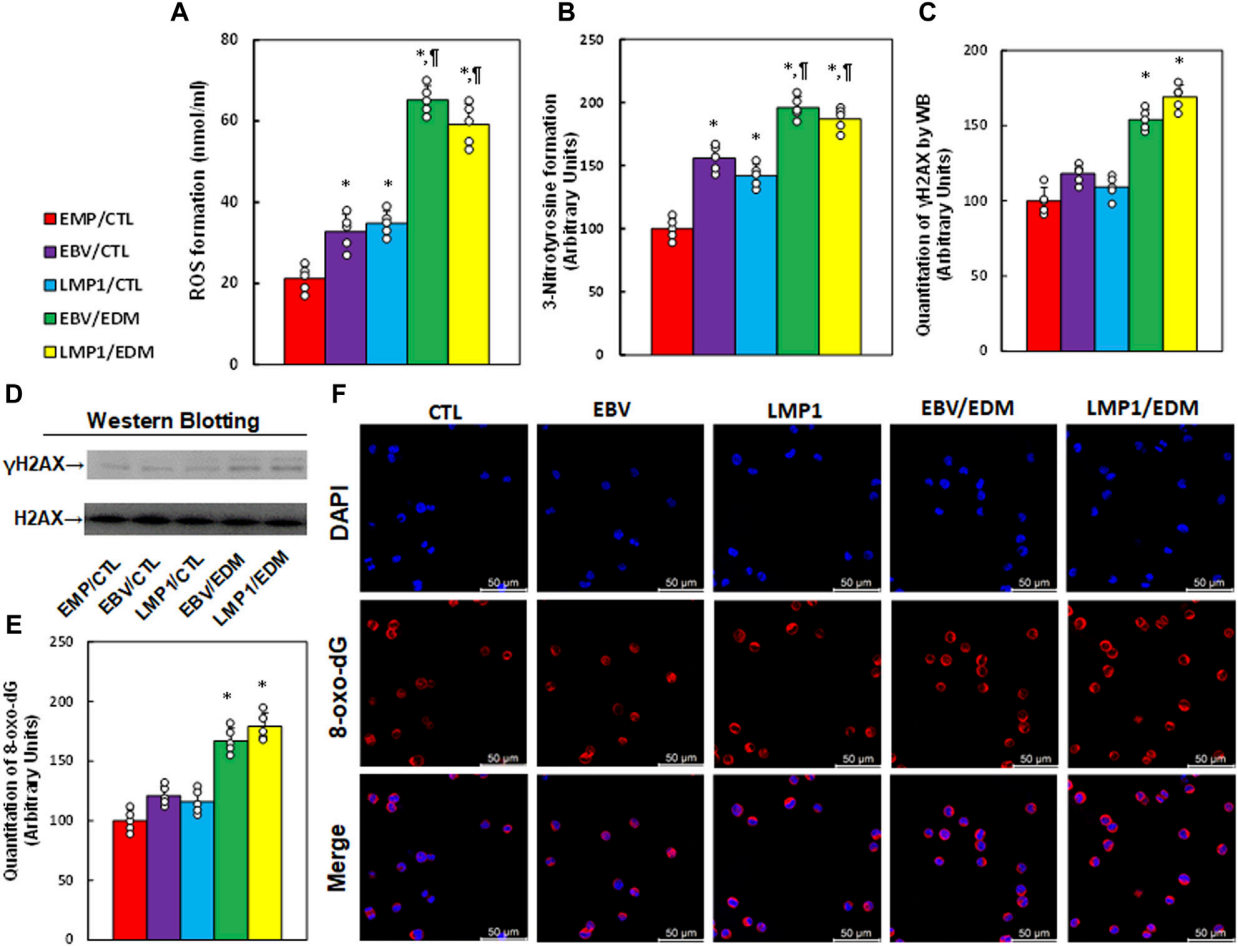
EDM modulates EBV exposure-mediated oxidative stress in HESC cells. Immortalized human HESC cells at passage 6 were pre-treated with empty (EMP), EBV or LMP1 adenovirus, then exposed to either control (CTL) or 4.0 µM of EDM for 48 h, then the cells were harvested for subsequent biological assays. **(A)** ROS formation. **(B)** 3-nitrotyrosine formation. **(C)** γH2AX quantitation. **(D)** Representative western blots for **(C)**. **(E)** Quantitation of 8-oxo-dG formation. **(F)** Representative pictures for **(E)**. n = 5, *p < 0.05, vs. EMP/CTL treatment; ¶*p* < 0.05, vs. EBV/CTL treatment. Data were expressed as mean ± SD, one-way ANOVA was used for statistical analysis. Abbreviations EBV, Epstein-Barr virus; EDM, evodiamine; ERα, estrogen receptor α; ERβ, estrogen receptor β; LMP1, latent membrane protein 1; 8-oxo-dG, 8-Hydroxy-2′-deoxyguanosine; ROS, reactive oxygen species; WB, Western blotting.

Immortalized human HESC cells underwent pre-treatment with EMP, EBV, or LMP1, followed by treatment with either CTL or 4.0 µM of EDM for 48 h at passage 6 for the measurement of oxidative stress. The findings indicated that pre-treatment with either EBV or LMP1 significantly augmented ROS formation (see Figure 3A) and 3-nitrotyrosine (see Figure 3B) in comparison to the EMP/CTL group. Interestingly, EDM treatment further intensified this effect. Conversely, pre-treatments with EBV/LMP1 had no notable impact on γH2AX formation (see Figures 3C, D; Supplementary Figure S1C) and 8-oxo-dG generation (see Figures 3E, F) in comparison to the EMP/CTL group. However, EDM treatment significantly increased the formation of both γH2AX and 8-oxo-dG. In conclusion, EDM treatment modulates EBV exposure-mediated redox balance in HESC cells.

### EDM ameliorates EBV exposure-mediated pro-inflammation in HESC cells

We examined the impact of EDM treatment and transient exposure to EBV/LMP1 on the release of pro-inflammatory cytokines in HESC cells. It was noted that pre-treatment with either EBV or LMP1 significantly elevated mRNA levels of these pro-inflammatory cytokines. EDM treatment partially restored the expression of IL1β and PGE2 but completely restored the expression of IL6 and TNFα (see Supplementary Figure S4A). We also assessed the cellular secretion of these cytokines, including IL1β (see Supplementary Figure S4B), IL6 (see Supplementary Figure S4C), TNFα (see Supplementary Figure S4D), and PGE2 (see Supplementary Figure S4E), and the secretion pattern mirrored that of the mRNA levels.

### EDM modulates mitochondrial function in HESC cells influenced by transient exposure to EBV

**FIGURE 4:**
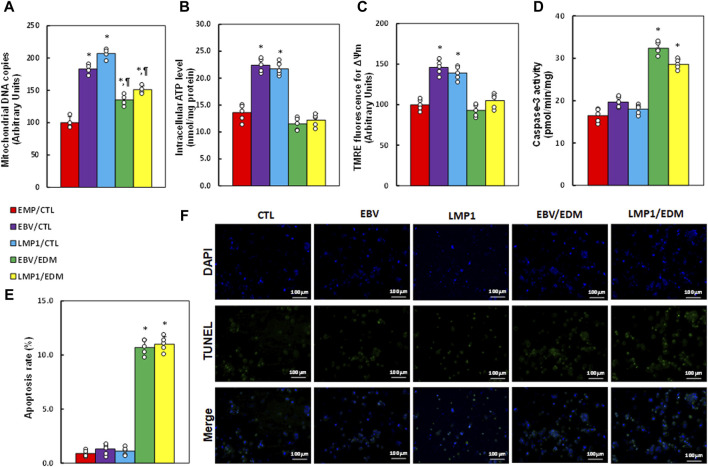
EDM modulates EBV exposure-mediated mitochondrial function in HESC cells. Immortalized human HESC cells at passage 6 were pre-treated with empty (EMP), EBV or LMP1 adenovirus, then exposed to either control (CTL) or 4.0 µM of EDM for 48 h, then the cells were harvested for subsequent biological assays. **(A)** Mitochondrial DNA copies. **(B)** Intracellular ATP levels. **(C)** Mitochondrial membrane potential (ΔѰm). **(D)** Caspase-3 activity. **(E)** Apoptosis rate. **(F)** Representative pictures for **(E)**. n = 5. *p < 0.05, vs. EMP/CTL treatment; ¶*p* < 0.05, vs. EBV/CTL treatment. Data were expressed as mean ± SD, one-way ANOVA was used for statistical analysis. Abbreviations ATP, Adenosine triphosphate; EBV, Epstein-Barr virus; EDM, evodiamine; LMP1, latent membrane protein 1; ROS, reactive oxygen species; TUNEL, terminal deoxynucleotidyl transferase dUTP nick end labeling.

We investigated the impact of EDM treatment and transient EBV/LMP1 exposure on mitochondrial function. Results revealed that pre-treatment with either EBV or LMP1 significantly enhanced mitochondrial replication (see Figure 4A), intracellular ATP levels (see Figure 4B), and mitochondrial membrane potential (see Figure 4C; Supplementary Figure S5) compared to the EMP/CTL group. EDM treatment either partially or completely reversed this effect. Additionally, EBV/LMP1 pre-treatment had minimal impact on caspase-3 activity (see Figure 4D) and apoptosis rate (see Figures 4E, F), while EDM treatment significantly elevated these levels compared to the EMP/CTL group.

### EDM suppresses EBV exposure-mediated cell proliferation in HESC cells

**FIGURE 5:**
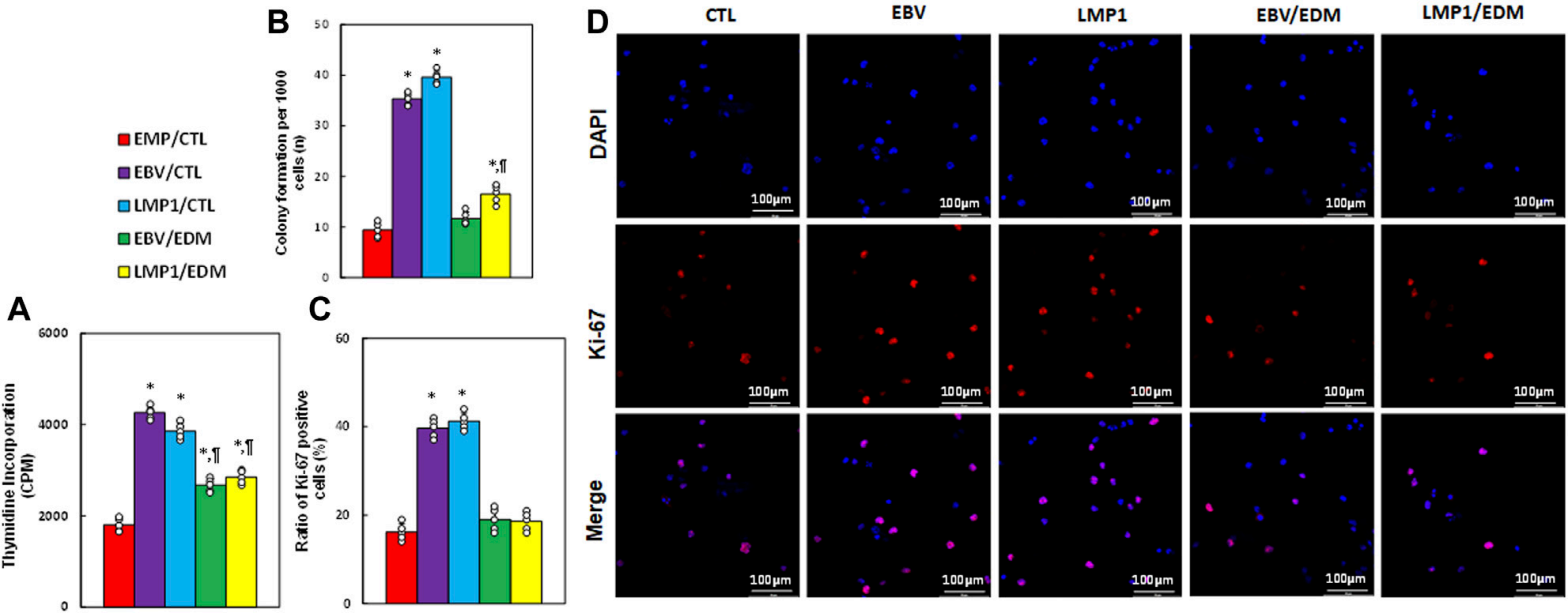
EDM suppresses EBV exposure-mediated cell proliferation in HESC cells. Immortalized human HESC cells at passage 6 were pre-treated with empty (EMP), EBV or LMP1 adenovirus, then exposed to either control (CTL) or 4.0 µM of EDM for 48 h, then the cells were harvested for subsequent biological assays. **(A)** Thymidine incorporation (CPM). **(B)** Colony formation. **(C)** Ratio of Ki67 positive cells. **(D)** Representative pictures for **(C)**. n = 5. *p < 0.05, vs. EMP/CTL treatment; ¶*p* < 0.05, vs. EBV/CTL treatment. Data were expressed as mean ± SD, one-way ANOVA was used for statistical analysis. Abbreviations EBV, Epstein-Barr virus; EDM, evodiamine; LMP1, latent membrane protein 1.

We examined the impact of EDM treatment and transient exposure to EBV/LMP1 on cell proliferation. Results indicated that pre-treatment with either EBV or LMP1 significantly elevated thymidine incorporation (see Figure 5A), colony formation (see Figure 5B), and the ratio of Ki67-positive cells (see Figures 5C, D) in HESC cells compared to the EMP/CTL group. EDM treatment either partially or completely reversed this effect.

### EDM modulates redox balance and proinflammatory cytokine release in peripheral blood in LMP1-mediated endometriosis mouse model

**FIGURE 6:**
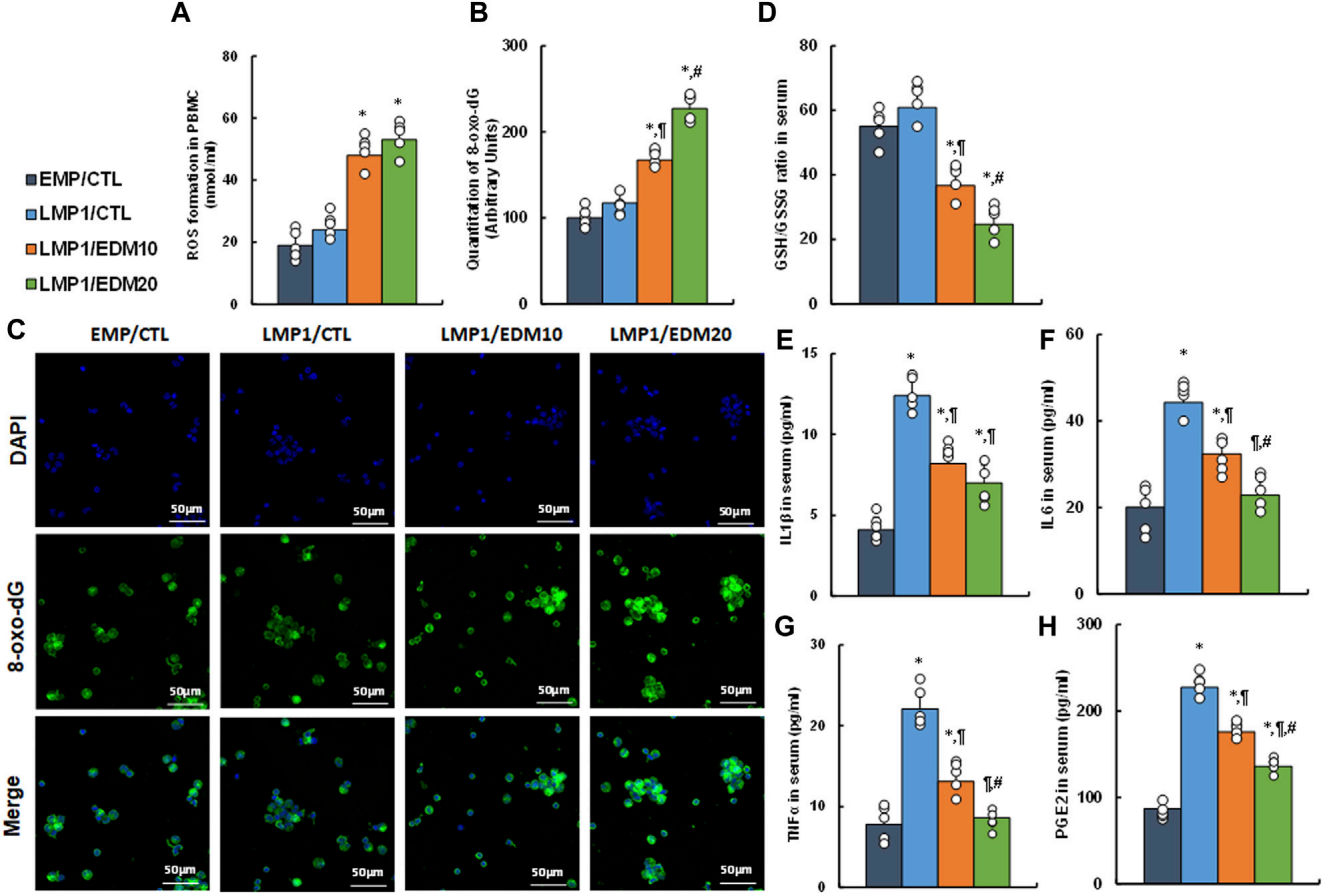
EDM modulates redox balance and proinflammatory cytokine release in peripheral blood in LMP1-mediated endometriosis mouse model. The mixture of immortalized human HESC and HEEC cells were pre-treated with either empty control (EMP) or LMP1 adenovirus, then cells were administered intraperitoneally, and the experimental mice were then treated by control (CTL), 10 mg/kg of EDM (EDM10) or 20 mg/kg of EDM (EDM20), then the serum and PBMC were isolated for biological assays. **(A–C)** PBMC cells were isolated for analysis. **(A)** ROS formation; **(B)** Quantitation of 8-oxo-dG formation; **(C)** Representative pictures for **(B)**. **(D–H)** The serum was isolated for analysis. **(D)** GSH/GSSG ratio in serum; **(E)** IL1β level **(B)**; **(F)** IL6 level; **(G)** TNFα level; **(H)** PGE2 level. n = 7. *p < 0.05, vs. EMP/CTL group; ¶*p* < 0.05, vs. LMP1/CTL group; #*p* < 0.05, vs. LMP1/EDM10 group. Data were expressed as mean ± SD, one-way ANOVA was used for statistical analysis. Abbreviations EBV, Epstein-Barr virus; EDM, evodiamine; ERβ, estrogen receptor β; GSH, glutathione; IL6, interleukin-6; IL1β, interleukin-1β; LMP1, latent membrane protein 1; 8-oxo-dG, 8-Hydroxy-2′-deoxyguanosine; PBMC, peripheral blood mononuclear cells; PGE2, Prostaglandin E2; TNFα, Tumor Necrosis Factor- α; ROS, reactive oxygen species.

A mixture of LMP1-pretreated HESC and HEEC cells was intraperitoneally administered as an endometriosis model and treated with either 10 mg/kg of EDM (EDM10) or 20 mg/kg of EDM (EDM20). PBMC and serum were isolated for biological assays. Initial assessment of redox balance in PBMC showed that LMP1 pre-treatment had minimal effect on ROS formation (see Figure 6A) and 8-oxo-dG formation (see Figures 6B, C) in PBMC, whereas EDM treatments significantly intensified these effects compared to the EMP/CTL group. Subsequent evaluation in the serum demonstrated that LMP1 pre-treatment had marginal impact, whereas EDM treatments significantly reduced the GSH/GSSG ratio compared to the EMP/CTL group; notably, EDM20 treatment exhibited a stronger effect than the EDM10 group (see Figure 6D). Moreover, LMP1 pre-treatment significantly increased proinflammatory cytokine release, including IL1β (see Figure 6E), IL6 (see Figure 6F), TNFα (see Figure 6G), and PGE2 (see Figure 6H), compared to the EMP/CTL group. EDM10 treatment partially, and EDM20 treatment almost completely (except for IL1β), reversed this effect.

### EDM suppresses ERβ expression and cell growth in LMP1-mediated endometriosis mouse model

**FIGURE 7:**
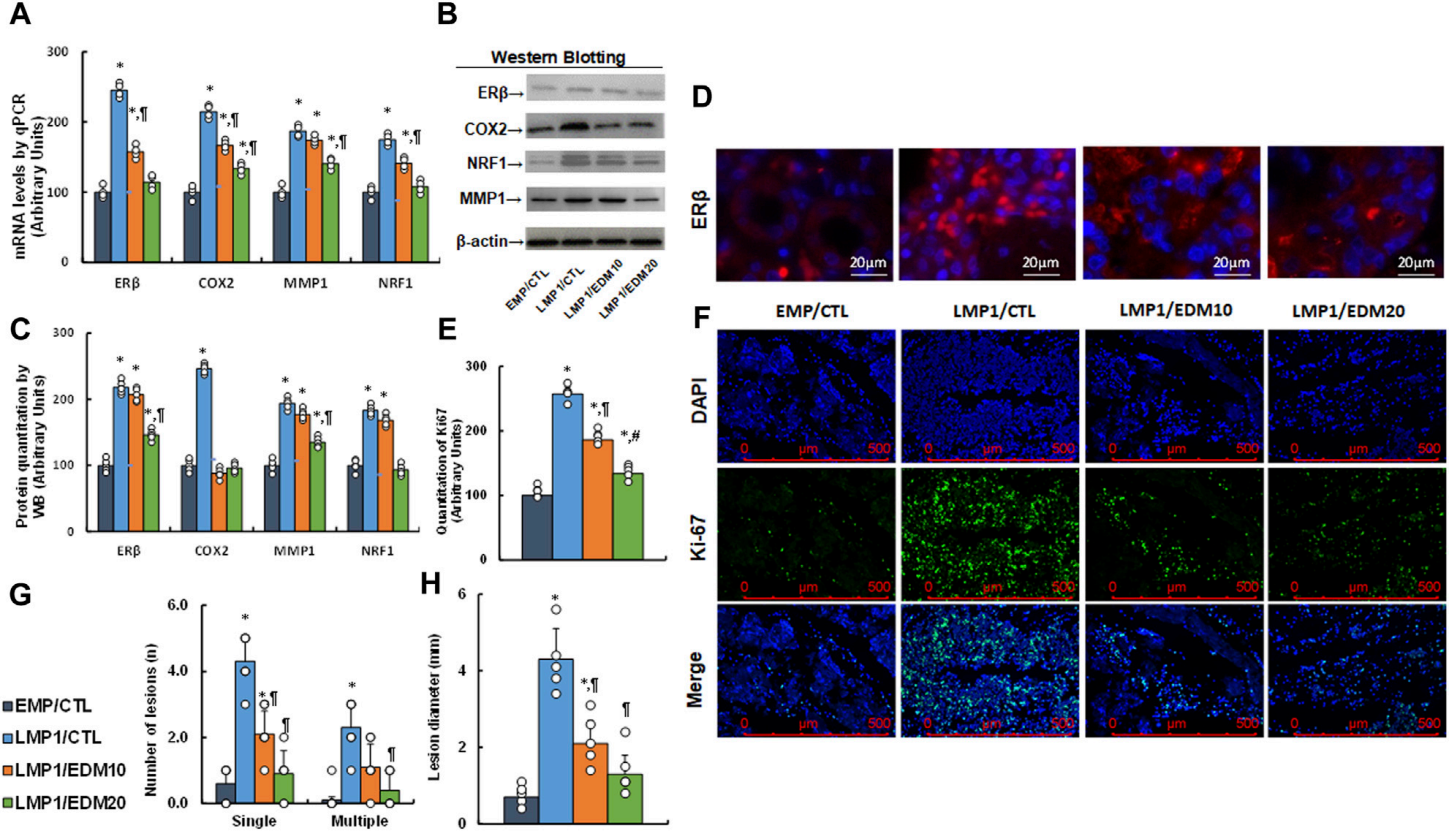
EDM suppresses ERβ and cell growth in LMP1-mediated endometriosis mouse model. The mixture of immortalized human HESC and HEEC cells were pre-treated with either empty control (EMP) or LMP1 adenovirus, then cells were administered intraperitoneally, and the experimental mice were then treated by control (CTL), 10 mg/kg of EDM (EDM10) or 20 mg/kg of EDM (EDM20), then the endometriosis tissues were isolated for biological assays. **(A)** mRNA levels by qPCR, n = 4. **(B)** Representative western blots. **(C)** Quantitation of protein levels for **(B)**, n = 5. **(D)** Representative pictures for ERβ staining. **(E)** Quantitation of Ki67, n = 7. **(F)** Representative pictures for **(E)**. **(G)** Number of lesions, n = 7. **(H)** Lesion diameter, n = 7. *p < 0.05, vs. EMP/CTL group; ¶*p* < 0.05, vs. LMP1/CTL group; #*p* < 0.05, vs. LMP1/EDM10 group. Data were expressed as mean ± SD, one-way ANOVA was used for statistical analysis. Abbreviations EBV, Epstein-Barr virus; EDM, evodiamine; COX2, cyclooxygenase 2; ERβ, estrogen receptor β; LMP1, latent membrane protein 1; NRF1, nuclear respiratory factor-1; MMP1, matrix metalloproteinase-1; WB, Western blotting.

A LMP1-pretreated mixture of HESC and HEEC cells was administered intraperitoneally as an endometriosis model and treated with either 10 mg/kg of EDM (EDM10) or 20 mg/kg of EDM (EDM20). Endometriosis tissues were isolated for biological assays. Gene expression analysis revealed that pretreatment with LMP1 markedly increased the mRNA levels of ERβ and the genes it regulates, such as COX2, MMP1, and NRF1. EDM20 treatment partially reversed this effect, with EDM20 exhibiting a more pronounced impact than the EDM10 group (see Figure 7A). Protein levels for these genes mirrored the expression pattern observed at the mRNA level (see Figures 7B, C; Supplementary Figure S1D). Immunohistochemistry (IHC) staining for ERβ expression demonstrated a similar pattern to that observed at the mRNA level (see Figure 7D). Furthermore, Ki67 expression assessed by IHC in these tissues revealed that LMP1 pretreatment significantly increased Ki67 staining compared to the EMP/CTL group. EDM10 treatment partially reversed this effect, while the EDM20 group exhibited a more substantial impact than the EDM10 group (see Figures 7E, F). Finally, assessment of endometriosis growth demonstrated that LMP1 pretreatment significantly increased the number of lesions (including both single and multiple lesions) (see Figure 7G) and the diameter of lesions (see Figure 7H) compared to the EMP/CTL group. EDM10 treatment partly reversed this effect, with the EDM20 group displaying a more pronounced impact than EDM treatment. In conclusion, EDM treatment inhibits LMP1 pretreatment-mediated endometriosis development in the mouse model.

### Evodiamine inhibits early EBV/LMP1 exposure-mediated later endometriosis development through ERβ suppression

**FIGURE 8:**
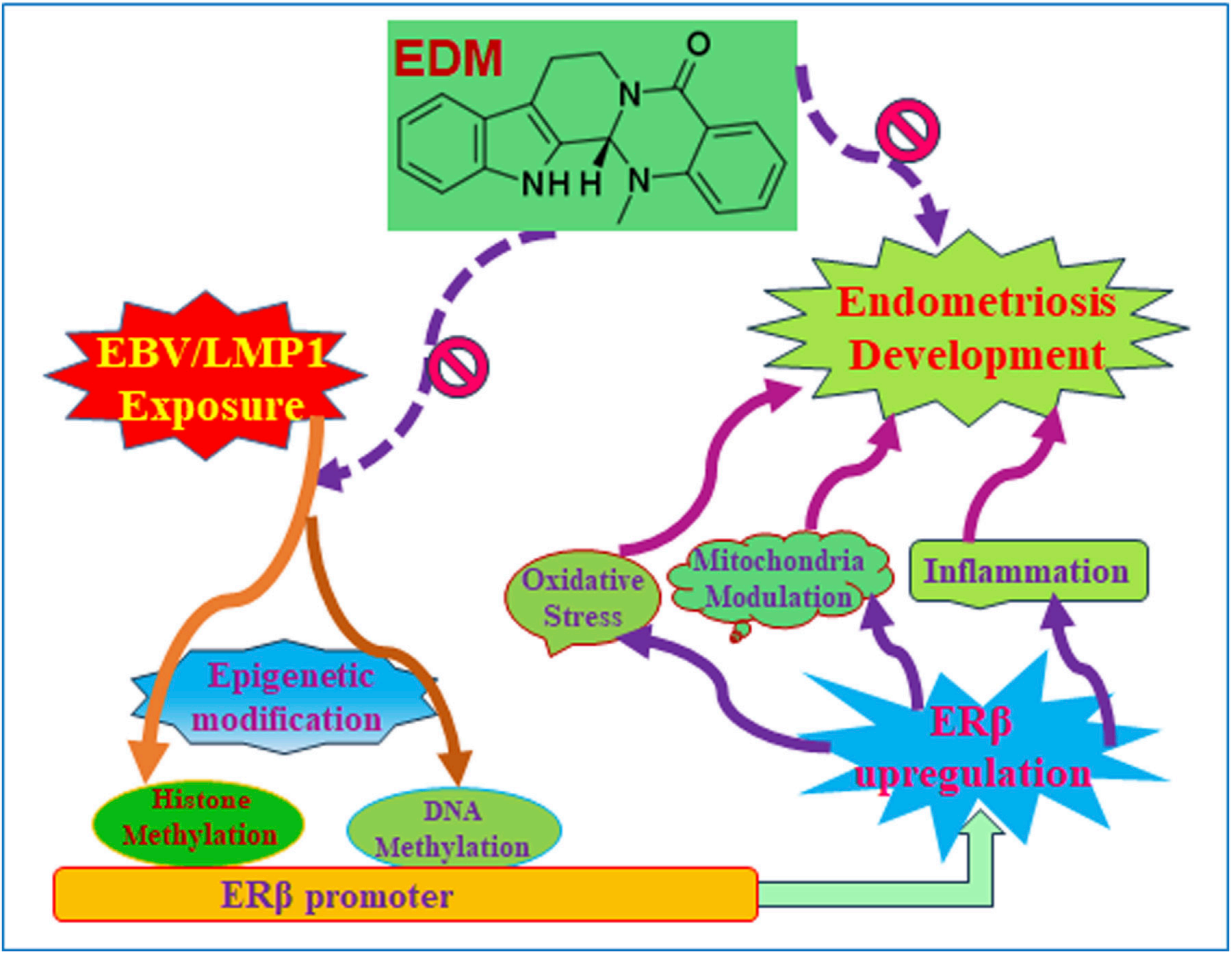
Evodiamine suppresses early EBV/LMP1 exposure-mediated later endometriosis development. Abbreviations EBV, Epstein-Barr virus; EDM, evodiamine; ERβ, estrogen receptor β; LMP1, latent membrane protein 1.

We developed a schematic model illustrating the potential impact of evodiamine on early exposure-mediated endometriosis induced by EBV/LMP1. Early exposure to either EBV or LMP1 induces epigenetic modifications on the ERβ promoter, including DNA methylation and histone methylation, and subsequently results in persistent ERβ upregulation even in the absence of EBV/LMP1. Increased ERβ expression triggers oxidative stress, mitochondrial modulation, and proinflammatory cytokine release, which then eventually triggers cell growth and endometriosis development. Conversely, evodiamine has the ability to impede the epigenetic alterations induced by EBV/LMP1 on the ERβ promoter, consequently suppressing the development of endometriosis initiated by EBV/LMP1 pretreatment (see Figure 8).

## Discussion

In this investigation, we discovered that brief exposure to either EBV or LMP1 leads to enduring ERβ expression via epigenetic modifications in HESC or HEEC cells even after the removal of EBV or LMP1. EDM treatment reverses the epigenetic changes induced by EBV/LMP1, subsequently affecting ERβ expression and the development of endometriosis, evidenced in both *in vitro* and *in vivo* studies involving mice.

### Involvement of EBV in the development of EMS

We demonstrate that temporary infection with either EBV or LMP1 induces epigenetic alterations on the ERβ promoter, leading to sustained upregulation of ERβ in both HESC and HEEC cells even in the absence of EBV or LMP1. This subsequently contributes to EMS development. LMP1, as an EBV-encoded oncogene (Chou et al., 2011), plays a critical regulatory role in EBV-mediated tumor metabolism (Wang et al., 2017). However, our study shows the potential contribution of early infection with EBV or LMP1 during childhood to later EMS development through epigenetic modifications and ERβ activation. This is a novel mechanism for the etiology of EMS, even though active EBV may not be readily detectable in the majority of EMS patients.

### Role of ERβ in EMS development

We demonstrated that ERβ expression undergoes persistent upregulation in the development of endometriosis mediated by transient exposure to EBV/LMP1. This upregulation extends to its target genes, including COX2 (Su et al., 2009), MMP1 (Hudelist et al., 2005) and NRF1 (Klinge, 2020). These genes subsequently influence cell metabolism, encompassing cell proliferation, inflammation, mitochondrial function, and redox balance, ultimately promoting the development of endometriosis. Our discoveries indicate that the expression of ERβ may have a crucial role (Bulun et al., 2012), it is important to note that other factors may also contribute to this process, as suppressing ERβ only partially inhibits, but does not completely halt, endometriosis development. This presents a potential therapeutic strategy for treating endometriosis through targeted ERβ suppression.

### Role of EDM in EMS treatment

Our *in vitro* cell culture study in HESC and HEEC cells revealed that treatment with 4 µM of EDM efficiently eliminates epigenetic modifications on the ERβ promoter induced by EBV/LMP1 exposure, consequently reversing ERβ expression and related molecular metabolism changes. Furthermore, our *in vivo* study demonstrated that administering 20 mg/kg body weight of EDM effectively suppresses EBV/LMP1-mediated endometriosis development in a mouse model, indicating an intriguing therapeutic potential for EDM in clinical treatment for endometriosis (Shyu et al., 2006; Shi et al., 2016).

## Conclusion

Transient exposure to EBV or LMP1 triggers epigenetic modifications, resulting in permanent ERβ upregulation and subsequent EMS development. EDM treatment can suppress EMS development through ERβ suppression. This study unveils a novel mechanism for the development of endometriosis later in adulthood, stemming from early latent exposure to EBV during childhood. Furthermore, EDM emerges as an encouraging option for the clinical management of endometriosis.

## Data Availability

The original contributions presented in the study are included in the article/Supplementary Material, further inquiries can be directed to the corresponding authors.

## Abbreviations

ChIP, chromatin immunoprecipitation; EBV, Epstein-Barr virus; EDM, evodiamine; COX2, cyclooxygenase 2; EMS, Endometriosis; ERβ, estrogen receptor β; GSH, glutathione; HEEC, human endometrial epithelial cells; HESC, human endometrial stromal cells; IHC, immunohistochemistry; IL6, interleukin-6; IL1β, interleukin-1β; LMP1, latent membrane protein 1; NRF1, nuclear respiratory factor-1; 8-oxo-dG, 8-Hydroxy-2′-deoxyguanosine; PBMC, peripheral blood mononuclear cells; PGC1β, peroxisome proliferator-activated receptor gamma coactivator 1-β; PGE2, Prostaglandin E2; MMP1, matrix metalloproteinase-1; TNFα, Tumor Necrosis Factor- α; ROS, reactive oxygen species.
