# Supplementary material for: Evodiamine suppresses endometriosis development induced by early EBV exposure through inhibition of ERβ
Source: Front Pharmacol. 2024 Aug 1;15:1426660. doi: 10.3389/fphar.2024.1426660 (PMC11324466; doi:10.3389/fphar.2024.1426660)
Supplement: Supplementary file 1 [file DataSheet1.PDF]

Evodiamine Suppresses Endometriosis Development Induced by Early EBV Exposure through Inhibition of ERβ

Junling Wang[1], Yuanqi Liang[2], Xiaoru Liang[2], Huijuan Peng[1], Yongxia Wang[1], Mingtao Xu[1], Xuefang Liang[1], Helen Yao[3], Xiaohan Liu[4], Liqin Zeng[4], Paul Yao[1,#], Dongfang Xiang[1,#]

Supplementary Materials

Data S1: Materials and Methods

Reagents and materials. The human endometrial epithelial cells (HEEC, #ABC-H0045X) and human endometrial stromal cells (HESC, #ABI-TM257D) were immortalized by SV40 large T antigen, and purchased from AcceGen (Beijing, China), and cultured in medium using ABC-TM045X Human Endometrial Epithelial Cells Medium Kit and ABI-TM257D Human Endometrial Stromal Cell Line (HESC) Medium Kit, respectively (from AcceGen). The tumor cell line B95-8 and Namalwa were purchased from ATCC and cultured in RPMI 1640 medium containing 2mmol/l glutamine supplemented with 100 U/ml penicillin, 100 μg/ml streptomycin, 10% human serum and 1000 U/ml recombinant human IL-2. All cells were maintained in a humidified incubator with 5% CO_2 at 37°C. The B95-8 cell line was used for EBV viral production and subsequent concentration for the infection of cells according to the previous protocol (1). The EBV LMP1 adenovirus for LMP1 transient infection and related empty control adenovirus were provided by Dr. Haimou Zhang (from Hubei University).

Evodiamine (EDM), >99% purity, with molecular formula C19H17N3O, and molecular weight 303.36, was purchased from Wuhan Ability Chemical Technology Co. Ltd. China, and dissolved in dimethyl sulfoxide (DMSO, Sigma China) (2).

Antibodies for β-actin (sc-47778), COX2 (sc-19999), Ki-67 (sc-101861), MMP1 (sc-21731) and NRF1 (sc-101102) were obtained from Santa Cruz Biotechnology. Antibodies for H3K9me2 (ab1220), H3K9me3 (ab8898), H3K27me2 (ab24684), H3K27me3 (ab6002), H2AX (ab20669), γH2AX (ab2893) were obtained from Abcam. The antibodies for EBV LMP1 (NBP1-79009) and 8-oxo-dG (4354-MC-050) were obtained from Novus Biologicals. Nuclear extracts were prepared using the NE-PER Nuclear and Cytoplasmic Extraction Reagents Kit (Pierce Biotechnology). Protein concentration was measured using the Coomassie Protein Assay Kit (Pierce Biotechnology) per manufacturers' instructions.

RT reaction and real-time quantitative PCR. Total RNA from treated cells was extracted using the RNeasy Micro Kit (Qiagen), and the RNA was reverse transcribed using an Omniscript RT kit (Qiagen). All the primers were designed using Primer 3 Plus software with the Tm at 60°C, primer size of 21 bp, and product length in the range of 140-160 bp (see Table S1). The primers were validated with an amplification efficiency in the range of 1.9-2.1 and the amplified products were confirmed with agarose gel. Real-time quantitative PCR was run on iCycler iQ (Bio-Rad) using the Quantitect SYBR green PCR kit (Qiagen). The PCR was performed by denaturing at 95°C for 8 min followed by 45 cycles of denaturation at 95°C, annealing at 60°C, and extension at 72°C for 10 s, respectively. 1 μl of each cDNA was used to measure target genes. β-actin was used as the housekeeping gene for transcript normalization, and the mean values were used to calculate relative transcript levels with the $^{\Delta\Delta}$CT method per instructions from Qiagen. In brief, the amplified transcripts were quantified by the comparative threshold cycle method using β-actin as a normalizer. Fold changes in gene mRNA expression were calculated as $2^{-\Delta\Delta CT}$ with CT = threshold cycle, ΔCT=CT (target gene)-CT(β-actin), and the ΔΔCT =ΔCT (experimental)-ΔCT (reference) (3, 4).

Cell viability and MTT assay. Cells were pooled in 12-well plates, following exposure to different treatments at 80% confluence. Cell viability was analyzed by the MTT (3-(4,5-dimethylthianol-2-yl)- 2,5 diphenyltetrazolium bromide) reduction assay. Briefly, cells in each well were aspirated and washed with PBS, then 0.2ml of 0.3mg/ml MTT solution was added at 25°C for 3 hours. Thereafter, the precipitated blue formazan product was extracted by incubating samples with 0.1ml 10% SDS (dissolved by 0.01M HCl) overnight at 37°C. The optical density (OD) of formazan concentrations were determined at 560nm and the background was subtracted at 670nm, then normalized by cell numbers and expressed as OD/10^6 cells (5).

Chromatin immunoprecipitation (ChIP). Cells were washed and crosslinked using 1% formaldehyde for 20 min and terminated by 0.1M glycine. Cell lysates were sonicated and centrifuged. 500 µg of protein were pre-cleared by BSA/salmon sperm DNA with preimmune IgG and a slurry of Protein A Agarose beads. Immunoprecipitations were performed with the indicated antibodies, BSA/salmon sperm DNA and a 50% slurry of Protein A agarose beads. Input and immunoprecipitates were washed and eluted before then being incubated with 0.2 mg/ml Proteinase K for 2 h at 42˚C, followed by 6 h at 65˚C to reverse the formaldehyde crosslinking. DNA fragments were recovered through phenol/chloroform extraction and ethanol precipitation. A ~150 bp fragment on the related promoter was amplified by real-time PCR (qPCR) using the primers provided in Table S1 (3, 4).

Western blotting. Cells were lysed in an ice-cold lysis buffer (0.137M NaCl, 2mM EDTA, 10% glycerol, 1% NP-40, 20mM Tris base, pH 8.0) with protease inhibitor cocktail (Sigma). The proteins were separated in 10% SDS-PAGE and transferred to the PVDF membrane, which was then blotted using primary antibodies (1:1000) and then simultaneously incubated with the differentially labeled species-specific secondary antibodies, anti-RABBIT IRDye™ 800CW (green) and anti-MOUSE (or goat) ALEXA680 (red). Membranes were scanned and quantitated using the ODYSSEY Infrared Imaging System (LI-COR, NE) (6).

Immunostaining. The treated cells were transferred to cover slips coated with 0.1% gelatin, fixed by 3.7% formaldehyde at 37ºC for 15 min, permeabilized by 1% BSA+0.2% Triton X-100 in PBS for 1 hour, then blotted with 40 µg/ml (dilute 1:50) of either Ki-67 or 8-oxo-dG monoclonal antibodies for 2 hours. Cells were then washed three times and the FITC/Texas Red labeled anti-mouse/rabbit secondary antibody (1:100) was added for blotting for another 1 hour. After thorough washing, the slides were visualized and photographed; cell nuclei were stained with 4',6-diamidino-2-phenylindole dihydrochloride (DAPI, #D9542, from Sigma) and the staining was quantitated by Image J.

Measurement of oxidative stress. Treated cells were seeded in a 24-well plate and incubated with 10 µM CM-H2DCFDA (Invitrogen) for 45 min at 37°C; intracellular formation of reactive oxygen species (ROS) was then measured at excitation/emission wavelengths of 485/530nm using a FLx800 microplate fluorescence reader (Bio-Tek), and the data was normalized as arbitrary units (4, 7). The GSH/GSSG ratio was measured using the GSH/GSSG-Glo™ Assay Kit (#V6611, obtained from Promega) per manufacturers' instructions. 3-nitrotyrosine (3-NT) was measured using a 3-Nitrotyrosine ELISA Kit (#ab116691 from Abcam) according to manufacturers' instructions. The formation of γH2AX was measured from nuclear extracts by western blotting using H2AX as the input control (4).

Measurement of mitochondrial function.

Mitochondrial DNA copies. Genomic DNA was extracted from treated cells using a QIAamp DNA Mini Kit (Qiagen) and the mitochondrial DNA was extracted using the REPLI-g Mitochondrial DNA

Kit (Qiagen). The purified DNA was used for the analysis of genomic β-actin (marker of the nuclear gene) and ATP6 (ATP synthase F0 subunit 6, marker of the mitochondrial gene) respectively using the qPCR method as mentioned above. The primers for genomic β-actin are as follows: forward 5'- ctg gac ttc gag caa gag atg -3' and reverse: 5'- agg aag gaa ggc tgg aag agt -3'. Primers for ATP6: forward 5'- cat tta cac caa cca ccc aac -3' and reverse 5'- tat ggg gat aag ggg tgt agg -3'. The mitochondrial DNA copies were obtained from relative ATP6 copies that were normalized by β-actin copies using the $^{\Delta\Delta}$CT method (3, 7, 8).

Intracellular ATP level. The intracellular ATP level was determined using the luciferin/luciferase-induced bioluminescence system. An ATP standard curve was generated at concentrations of 10^{-12}-10^{-3}M. Intracellular ATP levels were calculated and expressed as nmol/mg protein (3, 7, 8).

Mitochondria membrane potential. Mitochondrial membrane potential (MMP, Δψm) was measured using TMRE (from Molecular Probes T-669) staining. A 600 μM T-669 stock solution was prepared using DMSO. Cells were grown on coverslips and immersed in 600 nM TMRE for 20 min at 37°C to load them with dye. The labeling medium was then aspirated and the cells were immersed in 150 nM TMRE to maintain the equilibrium distribution of the fluorophore. The coverslips were mounted with live cells onto confocal microscopes to image the cells using 548 nm excitation/573 nm emission filters. The intensity of TMRE fluorescence was measured using Image J software. Data from 10-20 cells were collected for each experimental condition and mean values of fluorescence intensity ± SEM were calculated (4, 8, 9).

Measurement of apoptosis. Apoptosis was evaluated by TUNEL assay using the In Situ Cell Death Detection Kit™ (Roche). Cells were fixed in 4% paraformaldehyde and labeled with TUNEL reagents. Stained cells were photographed using a fluorescence microscope and further quantified by FACS analysis (7).

DNA methylation analysis. We developed a real-time PCR-based method for methylation-specific PCR (MSP) analysis on the human ERβ promoter according to the previously described method with some modifications (10, 11). Genomic DNA from treated human endometrial cells was extracted and purified before then being treated by bisulfite modification using the EpiJET Bisulfite Conversion Kit (#K1461, Fisher). The modified DNA was amplified using methylated and unmethylated primers for MSP that were designed using the Methprimer software: (http://www.urogene.org/cgi-bin/methprimer/methprimer.cgi) with the below details: Methylated primer: forward 5'- gga tta tag gcg tga gtt att acg t -3', reverse 5'- att taa aca caa aaa ttt aat cac gaa -3'; unmethylated primer: forward 5'- gga tta tag gtg tga gtt att atg t -3', reverse 5'- att taa aca caa aaa ttt aat cac aaa -3'. The product sizes were as follows: 210 bp (methylated) with Tm: 68.2°C & 210bp (unmethylated) with Tm: 67.6°C; The final methylation readout was normalized by unmethylated input PCR.

Detection of EBV copy number. Genomic DNA was extracted from treated tumor cell lines using a QIAamp DNA Mini Kit (Qiagen). The EBV DNA copy number was measured through qPCR using 50ng of total DNA with EBV BMRF1 primers (see Table S1), and the results were normalized using cellular β-actin (primers see Table S1) as an internal control (12, 13). The Namalwa cell line, which contains 2 EBV viral genome copies, was used as a standard to prepare calibration curves for both EBV BMRF1 and β-actin genes, and the EBV viral load was presented as the number of viral genomes per cell (14, 15).

Analysis of cytokines. Human cytokines, including IL1β, IL6, and TNF-α from in vitro cell culture supernatant, were measured using Human IL-1 beta/IL-1F2 Quantikine ELISA Kit (#DLB50), Human IL-6 Quantikine ELISA Kit (#D6050) and Human TNF-alpha Quantikine ELISA Kit (#DTA00D), respectively; Mouse cytokine secretion, including IL1β, IL6 and TNFα, were measured by Mouse IL-1β/IL-1F2 Quantikine ELISA Kit (#MLB00C), Mouse IL-6 Quantikine ELISA Kit (#M6000B) and Mouse TNF-alpha Quantikine ELISA Kit (#MTA00B), respectively; and

the PGE2 was measured by Prostaglandin E2 Parameter Assay Kit (#KGE004B) according to manufacturers' instructions from R&D Systems (16).

DNA synthesis by [^{3}H]-thymidine incorporation. Cell proliferation was evaluated as the rate of DNA synthesis by [^{3}H]-methylthymidine incorporation (17). Cells were pooled in 24-well plates until they reached 80% confluence. After treatment, cells were incubated with serum-free media containing ^{3}H-methylthymidine (0.5 µCi/well) for 2 hours and then washed twice with PBS. Cellular DNA was precipitated using 10% trichloroacetic acid and solubilized with 0.4M NaOH (0.5 ml/well). Incorporation of ^{3}H-methylthymidine into the DNA was measured in a scintillation counter and was determined as counts per minute (CPM) (4).

Colony formation in soft agar. This assay is a method for evaluating the ability of individual cell lines to grow in an anchorage-independent manner. Cells were resuspended in DMEM containing 5% FBS with 0.3% agarose and layered on top of 0.5% agarose in DMEM on 60mm plates. 1000 cells were seeded in 60mm soft agar dishes for 30 days. The dishes were examined twice per week, and colonies that grew beyond 50mm in diameter were scored as positive. Each experiment was done in quadruplicate (4).

In vivo mouse experiments. The NOD scid gamma (NSG) mouse was purchased from Jackson lab. All procedures involving mice were conducted in accordance with NIH regulations concerning the use and care of experimental animals and were approved by the Institutional Animal Care and Use Committee of Guangzhou University of Chinese Medicine. All the female mice were housed 4 or 5 per cage on a 12:12-h light-dark cycle and were given commercial rodent chow and water ad libitum on arrival. All animal handling was carried out under laminar-flow hoods, and all the invasive procedures were performed with the animals under isoflurane inhalation anesthesia or a cocktail of ketamine (100mg/kg) and xylazine (10mg/kg) intraperitoneally. At 4 weeks of age, the female mice received bilateral ovariectomy (OVX) surgery. After one week of surgery, all the NSG mice were implanted subcutaneously with sterile 60-day release pellets containing 0.72mg of 17β-estradiol (E2, Innovative Research of America, Shanghai China) via a ~3-mm incision on the dorsal aspect of the neck. After 2 days of E2 pellets administration, the mixed human endometrial cells were transplanted as described below:

<u>Cell transplantation and EDM treatment</u>. Immortalized HESC and HEEC cells were infected by either empty control or LMP1 adenovirus for 48 hours, then the adenovirus was removed and cultured continuously to pick up the single colony until passage 6. On the day of transplantation, LMP1 adenovirus or empty control (EMP)-treated immortalized HESC and HEEC cells were trypsinized and 2×10^6 of cells for both HESC and HEEC cells were mixed, pelleted and washed, then resuspended and mixed with Matrigel (BD Biosciences, Beijing China) at a 1:1 ratio in a final volume of 150µl, and maintained on ice until transplantation. The suspension mixtures were loaded in 1ml insulin syringe fitted with 20-gauge needle, and administered intraperitoneally on the midventral line just caudal to the umbilicus without any damage to the peritoneal layer or organs. Immediately after transplantation, the mice were kept to sternal posture to facilitate attachment of cells with peritoneum (18). After 24 hours of cell transplantation, the mice were randomly divided into below 4 groups and treated by EDM or equivalent vehicle. Group 1. cells were pretreated by empty adenovirus (EMP) for cell transplantation, then mice received vehicle control (EMP/CTL); Group 2. cells were pretreated by LMP1 for cell transplantation, then mice received vehicle control (LMP1/CTL); Group 3. cells were pretreated by LMP1 for cell transplantation, then mice received 10mg/kg body weight of EDM (EDM10) that was given as gavage every 2 days for 4 weeks (LMP1/EDM10); Group 4. cells were pretreated by LMP1 for cell transplantation, then mice received 20mg/kg body weight of EDM (LMP1/EDM20) (2).

Assessment and characterization of endometriosis lesions. After 4 weeks of cell transplantation, the recipient MSG mice were euthanized by CO2 asphyxiation, the whole blood was collected by heart puncture and the serum and peripheral blood mononuclear cells (PBMC) were isolated. The abdominal cavity was opened and the presence of endometriosis lesions was examined by gross visual examination. The number of endometriosis lesions was counted using a dissection microscope, and the size of each lesion was measured with a caliper. The lesions were categorized into single and multiple, where single lesions consisted of 1 visible nodule, and multiple lesions consisted of 2 or more visible nodules (18, 19). Part of the lesions were used for measurement of GSH/GSSG ratio, secretion of proinflammatory cytokines, gene expression was measured through real time PCR for mRNA and Western Blotting for protein levels (20). Immediately, the peritoneum was fixed in 4% paraformaldehyde, and the entire peritoneum was sectioned. Part of them were used for H&E staining, and the other sections were used for immunohistochemistry staining (18).

Isolation of mouse PBMC cells. Heparinized peripheral blood was collected from mouse subjects by heart puncture and diluted 1:3 with Hank's balanced salts solution without Ca^{2+}/Mg^{2+} (HBSS solution). The diluted blood was layered onto 10ml of Ficoll-Paque in 15ml sterile centrifuge tubes followed by centrifugation at 300×g at 20ºC for 40min. The PBMC layers were then harvested and washed by HBSS solution. The pellets were resuspended with lysing buffer containing 150 mM NH4Cl, 1.0 mM KHCO3, and 0.1 mM Na2EDTA, pH 7.4 and incubated for 5 min at room temperature to remove contaminated red cells. The cell suspensions were then centrifuged and washed with HBSS solution before the cell pellet was resuspended for further biological analysis.

Immunohistochemistry (IHC). The endometriotic lesions were cut into 10µm sections under Cryostat Microtome, and fixed in 2% paraformaldehyde for 15 min at room temperature and then fixed in methanol for 10 min at 4°C before being permeabilized by 1% BSA+0.2% Triton X-100 in PBS, and then blotted by 40µg/ml of antibodies for either Ki67 or ERβ for 2 hours. After another 1 hour of blotting by FITC labeled anti-mouse/rabbit secondary antibodies, the slides were visualized and photographed, and the protein expression (60 cells in each group) were quantitated by Image J. software (8, 19).

Statistical analysis. The data was given as mean ± SD; all of the experiments were performed at least in quadruplicate unless otherwise indicated. The data was analyzed as normal distribution using Shapiro-Wilk test to evaluate the normality of the data in SPSS 22 software (21), and the one-way analysis of variance (ANOVA) followed by the Tukey−Kramer test was used to determine statistical significance of different groups using SPSS 22 software, and a *P* value < 0.05 was considered significant (4).

REFERENCES

1. Lan K, Verma SC, Murakami M, Bajaj B, and Robertson ES. Epstein-Barr Virus (EBV): infection, propagation, quantitation, and storage. *Curr Protoc Microbiol.* 2007;Chapter 14:Unit 14E 2.
2. Yu Y, Huang X, Liang C, and Zhang P. Evodiamine impairs HIF1A histone lactylation to inhibit Sema3A-mediated angiogenesis and PD-L1 by inducing ferroptosis in prostate cancer. *Eur J Pharmacol.* 2023;957:176007.
3. Zou Y, Lu Q, Zheng D, Chu Z, Liu Z, Chen H, et al. Prenatal levonorgestrel exposure induces autism-like behavior in offspring through ERbeta suppression in the amygdala. *Mol Autism.* 2017;8:46.

4. Zhang H, Li L, Li M, Huang X, Xie W, Xiang W, et al. Combination of betulinic acid and chidamide inhibits acute myeloid leukemia by suppression of the HIF1alpha pathway and generation of reactive oxygen species. *Oncotarget.* 2017;8(55):94743-58.
5. Yao D, Li H, Gou Y, Zhang H, Vlessidis AG, Zhou H, et al. Betulinic acid-mediated inhibitory effect on hepatitis B virus by suppression of manganese superoxide dismutase expression. *Febs j.* 2009;276(9):2599-614.
6. Ceradini DJ, Yao D, Grogan RH, Callaghan MJ, Edelstein D, Brownlee M, et al. Decreasing intracellular superoxide corrects defective ischemia-induced new vessel formation in diabetic mice. *J Biol Chem.* 2008;283(16):10930-8.
7. Yao D, Shi W, Gou Y, Zhou X, Yee Aw T, Zhou Y, et al. Fatty acid-mediated intracellular iron translocation: a synergistic mechanism of oxidative injury. *Free Radic Biol Med.* 2005;39(10):1385-98.
8. Zhang H, Li L, Chen Q, Li M, Feng J, Sun Y, et al. PGC1beta regulates multiple myeloma tumor growth through LDHA-mediated glycolytic metabolism. *Mol Oncol.* 2018;12(9):1579-95.
9. Kong D, Zhan Y, Liu Z, Ding T, Li M, Yu H, et al. SIRT1-mediated ERbeta suppression in the endothelium contributes to vascular aging. *Aging Cell.* 2016.
10. Ogino S, Kawasaki T, Brahmandam M, Cantor M, Kirkner GJ, Spiegelman D, et al. Precision and performance characteristics of bisulfite conversion and real-time PCR (MethyLight) for quantitative DNA methylation analysis. *J Mol Diagn.* 2006;8(2):209-17.
11. Eads CA, Danenberg KD, Kawakami K, Saltz LB, Blake C, Shibata D, et al. MethyLight: a high-throughput assay to measure DNA methylation. *Nucleic Acids Res.* 2000;28(8):E32.
12. Verma D, Thompson J, and Swaminathan S. Spironolactone blocks Epstein-Barr virus production by inhibiting EBV SM protein function. *Proc Natl Acad Sci U S A.* 2016;113(13):3609-14.
13. Zuo L, Yu H, Liu L, Tang Y, Wu H, Yang J, et al. The copy number of Epstein-Barr virus latent genome correlates with the oncogenicity by the activation level of LMP1 and NF-kappaB. *Oncotarget.* 2015;6(38):41033-44.
14. Hui KF, and Chiang AK. Suberoylanilide hydroxamic acid induces viral lytic cycle in Epstein-Barr virus-positive epithelial malignancies and mediates enhanced cell death. *Int J Cancer.* 2010;126(10):2479-89.
15. Rose C, Green M, Webber S, Kingsley L, Day R, Watkins S, et al. Detection of Epstein-Barr virus genomes in peripheral blood B cells from solid-organ transplant recipients by fluorescence in situ hybridization. *J Clin Microbiol.* 2002;40(7):2533-44.
16. Kobayashi EH, Suzuki T, Funayama R, Nagashima T, Hayashi M, Sekine H, et al. Nrf2 suppresses macrophage inflammatory response by blocking proinflammatory cytokine transcription. *Nat Commun.* 2016;7:11624.
17. Somasundaram K, and El-Deiry WS. Inhibition of p53-mediated transactivation and cell cycle arrest by E1A through its p300/CBP-interacting region. *Oncogene.* 1997;14(9):1047-57.
18. Banu SK, Starzinski-Powitz A, Speights VO, Burghardt RC, and Arosh JA. Induction of peritoneal endometriosis in nude mice with use of human immortalized endometriosis epithelial and stromal cells: a potential experimental tool to study molecular pathogenesis of endometriosis in humans. *Fertil Steril.* 2009;91(5 Suppl):2199-209.
19. Arosh JA, Lee J, Balasubbramanian D, Stanley JA, Long CR, Meagher MW, et al. Molecular and preclinical basis to inhibit PGE2 receptors EP2 and EP4 as a novel nonsteroidal therapy for endometriosis. *Proc Natl Acad Sci U S A.* 2015;112(31):9716-21.
20. Gou Y, Li X, Li P, Zhang H, Xu T, Wang H, et al. Estrogen receptor beta upregulates CCL2 via NF-kappaB signaling in endometriotic stromal cells and recruits macrophages to promote the pathogenesis of endometriosis. *Hum Reprod.* 2019;34(4):646-58.

21. Ramezani G, Norouzi A, Arabshahi SKS, Sohrabi Z, Zazoli AZ, Saravani S, et al. Study of medical students' learning approaches and their association with academic performance and problem-solving styles. *J Educ Health Promot.* 2022;11:252.

Table S1. Sequences of primers for the real time quantitative PCR (qPCR)

Gene	Species	Analysis	Forward primer (5'→3')	Reverse primer (5'→3')
β-actin	Human	mRNA	gatgcagaaggagatcactgc	atactcctgcttgctgatcca
ERα	Human	mRNA	gggaagctactgtttgctcct	ttgaggcacacaaactcctct
ERβ	Human	mRNA	atgatgatgtccctgaccaag	acatcagccccatcattaaca
COX2	Human	mRNA	gcctgacacctttcaaattca	gaacattcctaccaccagcaa
MMP1	Human	mRNA	ttcccagcgactctagaaaca	ttcctgcatttgcttcaattt
NRF1	Human	mRNA	cgaggacacctcttacgatga	tcaaatacatgaggccgtttc
IL-1β	Human	mRNA	tgggataacgaggcttatgtg	gaacaccacttgttgctccat
IL-6	Human	mRNA	tccaaagatggctgaaaaaga	gctctggcttgttcctcacta
TNFα	Human	mRNA	tagcccatgttgtagcaaacc	aggacctgggagtagatgagg
PGE2	Human	mRNA	catcagttgagcactgcaaga	tctggcaaaactttcgaagaa
ERβ	Human	ChIP	ctcacattcccactcctctga	gaaacacagaagatattgccaag
β-actin	Human	Genome	tgtagcctgtacatcctccca	ggtctgcagtttgtacctgga
BMRF1	EBV	Genome	ggttttacaggtctggcatca	acccggggacttttatcttct
LMP1	EBV	mRNA	ccttcttcctagccatcatcc	ggtgttcatcagtgtgtcgtg

FIGURE S1

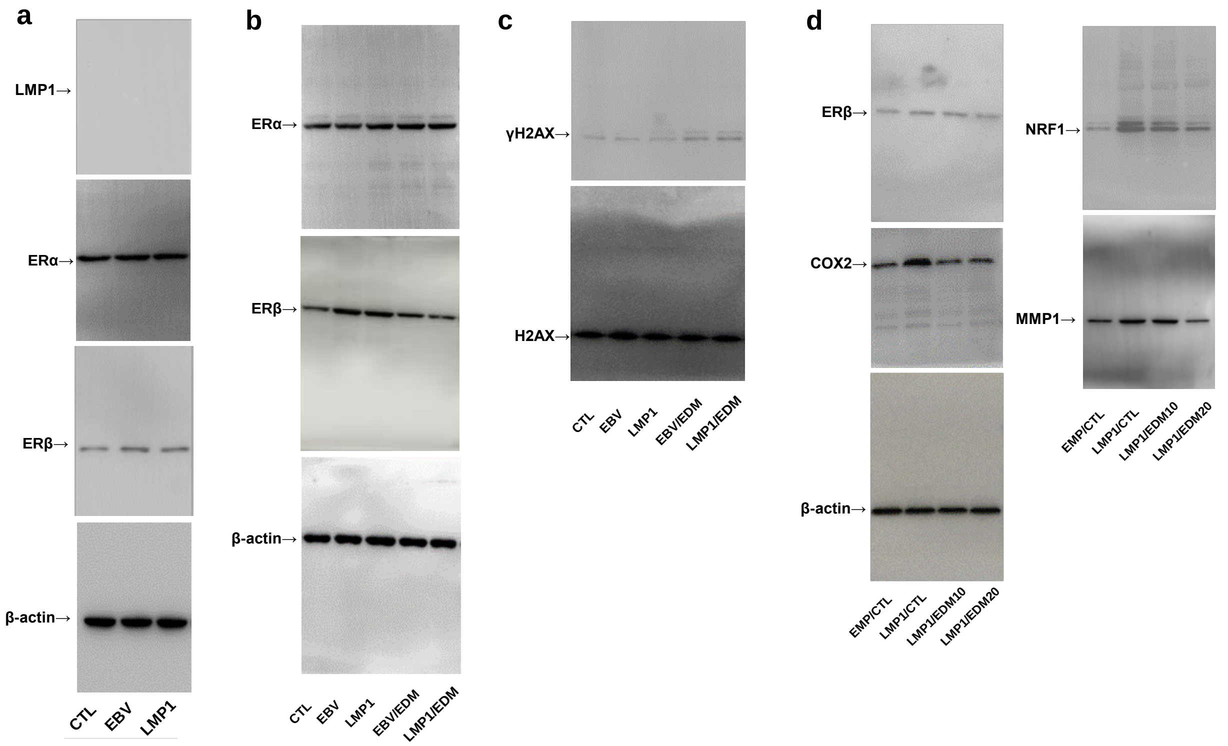

Figure S1. Representative pictures of full blots for Western Blotting. (a) Full blots for Figure 1f. (b) Full blots for Figure 2g. (c) Full blots for Figure 3d. (d) Full blots for Figure 7b

FIGURE S2

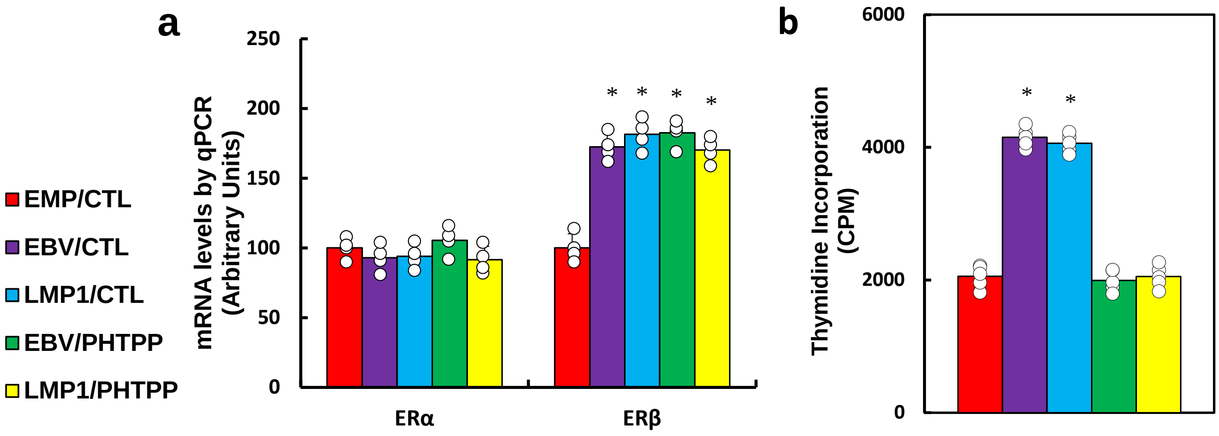

Figure S2. Transient exposure of EBV/LMP1- mediated ERβ activation can be diminished by ERβ specific blockade PHTPP. The empty (EMP), EBV or LMP1 adenovirus pre-treated immortalized human HESC cells at passage 6 were exposed to either control (CTL) or 5μM of ERβ blockade PHTPP for 48 hours for subsequent biological assays. (a) mRNA levels, n=4. (b) cell proliferation by thymidine incorporation, n=5. *, $P<0.05$, vs. EMP/CTL group.

FIGURE S3

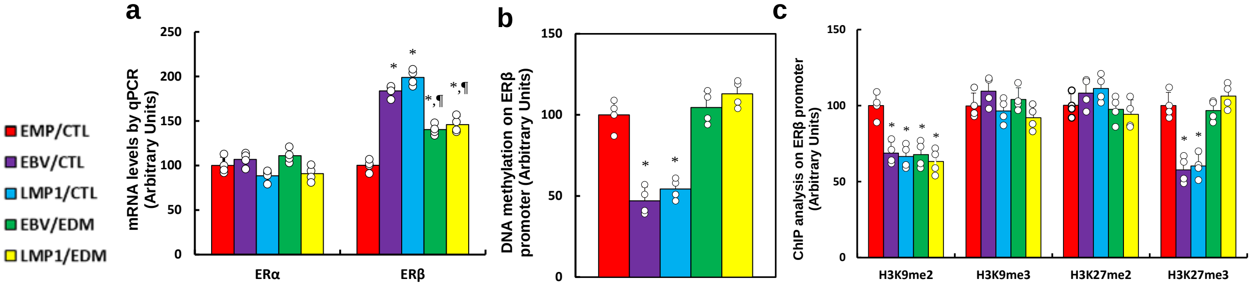

Figure S3. Transient exposure of EBV/LMP1 results in persistent ERβ upregulation in HEEC cells. The empty (EMP), EBV or LMP1 adenovirus pre-treated immortalized human HEEC cells at passage 6 were exposed to either control (CTL) or 4.0μM of EDM for 48 hours for subsequent biological assays. (a) mRNA levels (b) DNA methylation on ERβ promoter. (c) ChIP analysis on ERβ promoter. n=4, *, *P*<0.05, vs. CTL group.

FIGURE S4

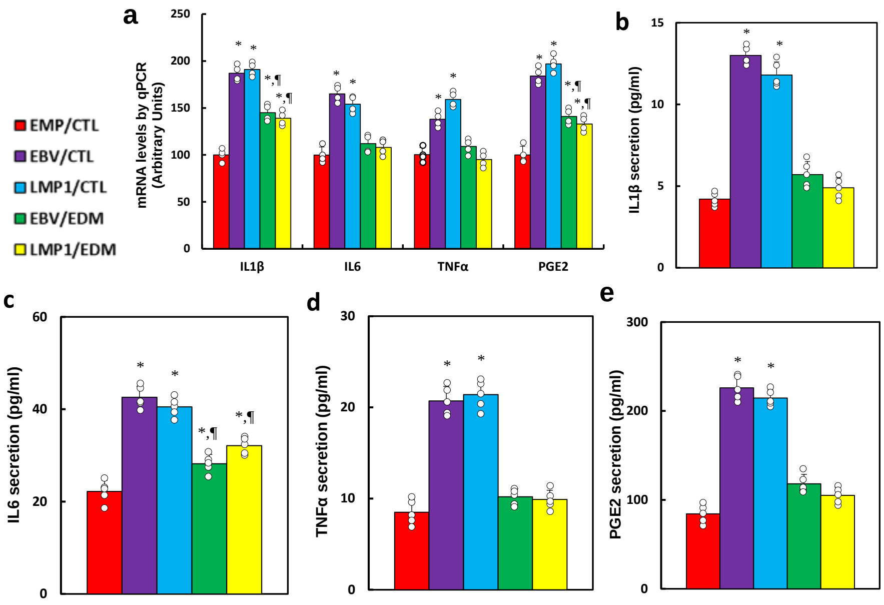

Figure S4. EDM ameliorates EBV exposure-mediated pro-inflammation in HESC cells. The empty (EMP), EBV or LMP1 adenovirus pre-treated immortalized human HESC cells at passage 6 were exposed to either control (CTL) or 4.0μM of EDM for 48 hours for biological assays. (a) mRNA levels, n=4. (b-e) Secretion of proinflammation cytokines, including IL1β (b), IL6 (c), TNFα (d) and PGE2 (e), n=5. *, *P*<0.05, vs. CTL treatment; ¶, *P*<0.05, vs. EBV treatment.

FIGURE S5

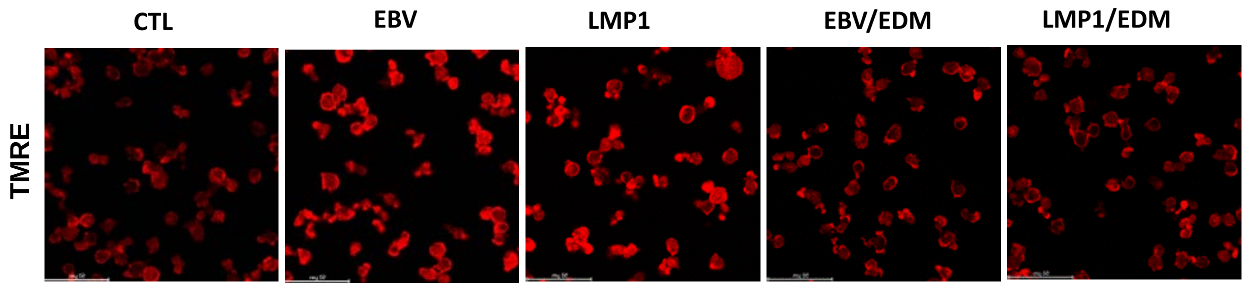

Figure S5. EDM modulates EBV/LMP1 exposure-mediated mitochondrial membrane potential in HESC cells. The empty (EMP), EBV or LMP1 adenovirus pre-treated immortalized human HESC cells at passage 6 were exposed to either control (CTL) or 4.0µM of EDM for 48 hours for analysis of mitochondrial membrane potential with representative TMRE staining pictures.
